# Bim Deletion Reduces Functional Deficits Following Ischemic Stroke in Association with Modulation of Apoptosis and Inflammation

**DOI:** 10.1007/s12017-022-08703-4

**Published:** 2022-02-11

**Authors:** Jason A. Glab, Hamsa Puthalakath, Shenpeng R. Zhang, Antony Vinh, Grant R. Drummond, Christopher G. Sobey, T. Michael De Silva, Hyun Ah Kim

**Affiliations:** 1 La Trobe Institute for Molecular Sciences, La Trobe University, Bundoora, VIC 3086 Australia; 2 Department of Microbiology, Anatomy, Physiology and Pharmacology, La Trobe University, Bundoora, VIC 3086 Australia

**Keywords:** Apoptosis, Stroke, Middle cerebral artery occlusion, Inflammation, Mouse

## Abstract

Cellular apoptosis is a key pathological mechanism contributing to neuronal death following ischemic stroke. The pro-apoptotic Bcl-2 family protein, Bim, is an important regulator of apoptosis. In this study we investigated the effect of Bim expression on post-stroke functional outcomes, brain injury and inflammatory mechanisms. Wild type (WT) and Bim-deficient mice underwent 1-h middle cerebral artery occlusion (MCAO) followed by 23 h of reperfusion. At 24-h post-stroke, we assessed functional deficit, infarct volume, immune cell death, as well as the number of infiltrating immune cells in the brain and circulating immune cells. Bim deficiency did not affect infarct volume (P > 0.05), but resulted in less motor impairment (~ threefold greater latency to fall in hanging grip strength test, P < 0.05) and a lower median clinical score than WT mice (P < 0.05). Additionally following MCAO, Bim-deficient mice exhibited fewer myeloid cells (particularly neutrophils) in the ischemic brain hemisphere and less apoptosis of CD3^+^ T cells in the spleen and thymus compared with WT (all P < 0.05). After MCAO, Bim-deficient mice also tended to have more M2-polarised macrophages in the brain than WT mice. In sham-operated mice, we found that Bim deficiency resulted in greater numbers of circulating total CD45^+^ leukocytes, Ly6C^lo+^ monocytes and CD3^+^ T cells, although MCAO did not affect the number of circulating cells at 24 h in either genotype. Our findings suggest that Bim deficiency modulates post-stroke outcomes, including reductions in motor impairment, brain inflammation and systemic post-stroke leukocyte apoptosis. Bim could therefore serve as a potential therapeutic target for stroke.

## Introduction

Ischemic stroke is a leading cause of death and disability worldwide. The clot-buster drug, recombinant tissue plasminogen activator, is still the only pharmacological therapy available for ischemic stroke and so a greater understanding of pathophysiological mechanisms in ischemic stroke is vital to identifying potential targets and develop novel therapies (Katan & Luft, 2018). Cell death, be it necrosis or apoptosis, following ischemic stroke is a key pathological mechanism contributing to infarct development. Whilst cells in the ischemic core die within minutes to hours of the event, those in the peri-infarct area may die several hours to days later (Deng et al., 2016; Radak et al., 2017). Although necrosis accounts for a large proportion of early neuronal death post-ischemia, apoptosis is an important cell death mechanism within the peri-infarct area, particularly at later stages (Sairanen et al., 2006). Apoptosis following ischemic stroke can be initiated by several stimuli, including excessive intracellular Ca^2+^, reactive oxygen species and DNA damage. This occurs mainly through the intrinsic mitochondrial pathway with additional contribution by the extrinsic death receptor-mediated pathway (Broughton et al., 2009). The intrinsic pathway of apoptosis is regulated by the Bcl-2 family proteins, consisting of anti- and pro-apoptotic members, which induce apoptosis when the balance between shifts toward the latter (Doerflinger et al., 2015).

Bcl-2-like protein 11, known as Bim, is a member of the pro-apoptotic BH3-only protein family (O'Connor et al., 1998). It is upregulated in response to a variety of stress stimuli, resulting in the activation of caspases through the intrinsic apoptotic pathway (Lee et al., 2013; Puthalakath et al., 2007). Bim is known to play a role in both physiological processes (e.g. embryonic development, T-cell selection) and disease states (e.g. diabetes mellitus, sepsis) (Doerflinger et al., 2015), including a key regulatory role in β-adrenoreceptor-mediated apoptosis (Lee et al., 2013). Indeed, deletion of Bim leads to an accumulation of lymphocytes due to a reduction in apoptosis (Chougnet et al., 2011). Specifically, Bim-deficient mice have increased numbers of memory T cells post-infection due to less apoptosis once the infection is resolved (Wojciechowski et al., 2006, 2007). Bim-knockout mice have improved outcomes in diseases, such as heart failure and sepsis (Doerflinger et al., 2016; Lee et al., 2013). Furthermore, depletion of CHOP, a transcriptional regulator of Bim, improves outcomes in a rat model of subarachnoid haemorrhage (He et al., 2012). However, the role of Bim in ischemic stroke has not been directly investigated.

It is well established that leukocytes enter the brain from the circulation after stroke. Subpopulations of these cells may exert either pro- or anti-inflammatory actions, resulting in opposing effects on the ongoing brain injury (Zhang et al., 2021). We and others have reported effects of several different leukocyte subtypes (e.g. monocytes, B cells, T cells) in the pathogenesis of ischemic stroke (Benakis et al., 2016; Chu et al., 2015, 2016; Hurn et al., 2007; Yilmaz et al., 2006). Whether Bim regulates the survival of pro- and/or anti-inflammatory leukocytes after stroke is unknown. Therefore, in the present study we have utilised Bim-deficient mice to determine the effect of Bim expression on post-stroke brain and systemic inflammation, brain injury and functional outcome measures.

## Methods

### Animals

All procedures were approved by the La Trobe University Animal Ethics Committee. Eight- to ten-week-old male C57Bl/6 wild type (WT; n = 38; 29 ± 6 g) and Bim-deficient (Bim^−/−^; n = 38; 26 ± 5 g) mice were used for experimentation. *Bim*^−/−^ mice were generated previously (Bouillet et al., 1999). Mice were excluded from the study if they (i) died during the surgical procedure (n = 1), (ii) experienced subarachnoid haemorrhage during intracerebral artery filament insertion (n = 12) or (iii) were euthanized due to not meeting inclusion criteria; i.e. < 65% reduction in regional cerebral blood flow (rCBF) during middle cerebral artery occlusion (MCAO) or < 50% recovery of rCBF within 5 min of reperfusion (n = 2).

### Middle Cerebral Artery Occlusion

Ischemia was induced through the occlusion of the right middle cerebral artery (MCA) by an intraluminal filament, as previously described (Kim et al., 2012). Mice were anesthetised by intraperitoneal injection of ketamine–xylazine (100 and 10 mg/kg, respectively) and body temperature was monitored by a rectal thermometer and maintained at 37.5 ± 0.5 °C using a heat lamp. To induce the ischemic event, the right proximal common carotid artery was clamped and a 6–0 nylon monofilament with a silicone-coated tip (Doccol Corporation) was inserted and advanced into the distal internal carotid artery and the Circle of Willis to occlude the origin of the MCA. The resulting occlusion of the MCA was confirmed by transcranial laser Doppler flowmetry (Perimed), where an approximately 80% reduction was observed in the area of the cerebral cortex supplied by the MCA. Once in position, the filament was tied in place and the clamp was removed to allow ischemia to persist for 1 h. The monofilament was then removed to allow for reperfusion for 23 h. Regional CBF (rCBF) levels were monitored for 30-min post-ischemia and reperfusion was confirmed by a return of rCBF to pre-ischemic levels within 5 min. The surgical wound was closed, and the animal was allowed to recover. Mice undergoing sham operation were anesthetised and their right carotid bifurcation exposed and separated from surrounding tissue, without the insertion of the monofilament. Post-surgery, all mice received 1 mL of 0.9% saline subcutaneously. Mice were provided with gel nectar (Able scientific), as well as their usual chow food and water, in individual cages on heat pads until euthanasia by CO_2_ asphyxiation the following day.

### Clinical Neurological Score Assessment

Prior to euthanasia, mice were clinically assessed by an investigator blinded to the experimental conditions and scored using a six-point scoring system. The scoring system was as follows: 0. normal motor function; 1. flexion of torso and contralateral forelimb when mouse is lifted by its tail; 2. circling when mouse is held by its tail on a flat surface; 3. leaning to one side at rest; 4. no spontaneous motor activity and 5. death within the 23-h reperfusion period. Additionally, a hanging grip test was performed to assess forelimb grip strength. Animals were suspended by their forelimbs on a wire at a height of 30 cm for up to 180 s, and the average latency to fall (s) from the wire from three trials interspaced by 5 min was calculated.

### Cerebral Infarct Volume

After euthanasia, mice were decapitated, brains were removed and immediately snap-frozen with liquid nitrogen. Thirty-micrometre-thick coronal sections separated by 420 µm were stained with 0.1% thionin to measure the infarct volume. Thionin-stained sections were imaged using TCapture (Version 5.1, Tucsen Photonics). Infarct volume is quantified using ImageJ software (NIH) using the following equation:

$${\text{CIV }} = \left[ {{\text{RIA }} - \left( {{\text{RHA }} - {\text{ LHA}}} \right)} \right]\times {{\text {thickness of slice}}},$$

where CIV is the corrected infarct volume, RIA is the right hemisphere infarct area, RHA is the right hemisphere area and LHA is the left hemisphere area.

Oedema volume is estimated according to the formula:

$${\text{EV }} = \left( {{\text{RHA }}{-}{\text{ LHA}}} \right) \, \times {\text{thickness of slice}}$$

where EV is the oedema volume.

### Flow Cytometry

Blood was collected by cardiac puncture, after which the mouse was intracardially perfused with PBS. The brain, spleen and thymus were then collected. Isolation of leukocytes from blood was performed using red blood cell lysis buffer (155 mM NH_4_Cl, 10 mM KHCO_3_, 3 mM EDTA). Spleen and thymus cell suspensions were made by mechanical dissociation through a 100 µm cell strainer into FACS buffer (1% bovine serum albumin in PBS), after which spleen cell suspensions were treated with red blood cell lysis buffer. Hemispheres of the brain were separated after removal of the cerebellum and olfactory bulb, which were mechanically dissociated in digestion buffer (125 U/ml collagenase type XI, 60 U/ml hyaluronidase, 450 U/ml collagenase type I-S in Ca^2+^/Mg^2+^-containing PBS) and incubated at 37 °C for 45 min in a shaking incubator (550 rpm). The suspension was passed through a 70 µm cell strainer and washed with PBS by centrifugation at 350 RCF and 4 °C for 10 min. After washing, the pellet was resuspended in 3 mL of 30% Percoll (GE Healthcare), beneath which an underlay of 2 mL 70% Percoll was pipetted and the sample was centrifuged at 1400 RCF at room temperature for 20 min with the brake off. The cells at the interphase of the Percoll concentrations were collected and washed in FACS buffer by centrifugation at 350 RCF for 10 min at 4 °C. Cells were counted and 1 × 10^6^ cells was used for subsequent analysis.

Cells are stained with the antibodies listed in Tables 1 and 2. Samples stained with the antibody panel in Table 1 are incubated with their respective combination on ice for 30 min. After their initial stain, all cells were stained with AlexaFluor 680-conjugated Annexin V (made in-house) for a further 1 h on ice. Following the second staining incubation, the cells were washed with FACS buffer by centrifugation at 485 RCF for 5 min at 4 °C. Samples stained with the antibody panel in Table 2 are first incubated with LIVE/DEAD™ Fixable Aqua Dead Cell Stain (Invitrogen) for 15 min at 4 °C. Cells were then washed with FACS buffer by centrifugation at 350 RCF and 4 °C for 5 min. An antibody cocktail containing those directed toward cell surface proteins (see Table 2) was then prepared, with 50 µL incubated on each sample for 25 min at 4 °C. The samples were again washed in FACS buffer by centrifugation. All cells were then fixed and permeabilised with FIX & PERM Cell Fixation & Cell Permeabilization Kit (Invitrogen), incubating them for 20 min at 4 °C. After incubation, the samples were washed with Permeabilization Wash (Invitrogen) diluted in dH_2_O by centrifugation. For intracellular staining, the FoxP3 antibody was diluted in Permeabilization Wash and applied to each of the samples and incubation at room temperature for 15 min. The samples were washed with Permeabilization Wash by centrifugation and resuspended in 1% formalin in FACS buffer. Cells were analysed using FACS Canto flow cytometer (BD Systems).

**Table 1:** Antibodies used for detection of apoptosis of immune cells in the spleen and thymus

Antigen	Tag	Target cells	Host/isotype	Clone	Supplier
F4/80	PE-Cy7	Macrophages	Rat IgG2a, κ	BM8	eBioscience
CD11b	AF488	B cells	Rat IgG2b	M1/70	BD Biosciences
Ly6G	PE	Neutrophils	Lewis IgG2a, κ	1A8	BD Biosciences
CD45R (B220)	V450	B cells	Rat IgG2a, κ	RA3-6B2	eBioscience
CD3e	FITC	T cells	Armenian Hamster IgG	145-2C11	eBioscience
CD4	PE	CD4+ T cells	Rat IgG2b, κ	GK1.5	eBioscience
CD8	BV510	CD8+ T cells	Louvain IgG2a, κ	3–6.7	BD Biosciences

**Table 2:** Antibodies used for flow cytometry

Antibody	Tag	Target cells	Host/isotype	Clone	Supplier
CD45	A700	Leukocytes	Rat IgG2b, κ	30-F11	BioLegend
CD3	APC	T cells	Armenian Hamster IgG	145-2C11	BioLegend
CD4	BV605	CD4+ T cells	Rat IgG2a, κ	RM4-5	BioLegend
FoxP3	PE-Cy5.5	Regulatory T cells	Rat IgG2a, κ	FJK-16s	eBioscience
CD11b	BV421	Myeloid cells	Rat IgG2b, κ	M1/70	BioLegend
CD206	PE	M2 macrophages	Rat IgG2a, κ	RA-6B2	BioLegend
F4/80-	APC-Cy7	Microglia/macrophages	Rat IgG2a, κ	BM8	BioLegend
Ly6C	FITC	Monocytes	Rat IgG2c, κ	HK1.4	BioLegend
Ly6G	PE-Cy7	Neutrophils	Rat IgG2a, κ	1A8	BioLegend

### Statistics

Outliers were removed from data using the ROUT method (Q = 1%). Statistical analyses of flow cytometry, spleen weight and thymus weight were performed using two-way ANOVA with Bonferroni multiple comparisons test between the means of relevant groups in R 4.1.0. All other experiments (except for the clinical score) were tested for statistical significance using an unpaired t test, assuming Gaussian distribution and equal variance between populations. Clinical score comparison was analysed using the Mann–Whitney test. Statistical significance was taken at P < 0.05.

## Results

### Bim-Deficient Mice have Less Severe Functional Outcomes After Stroke

**Fig. 1:**
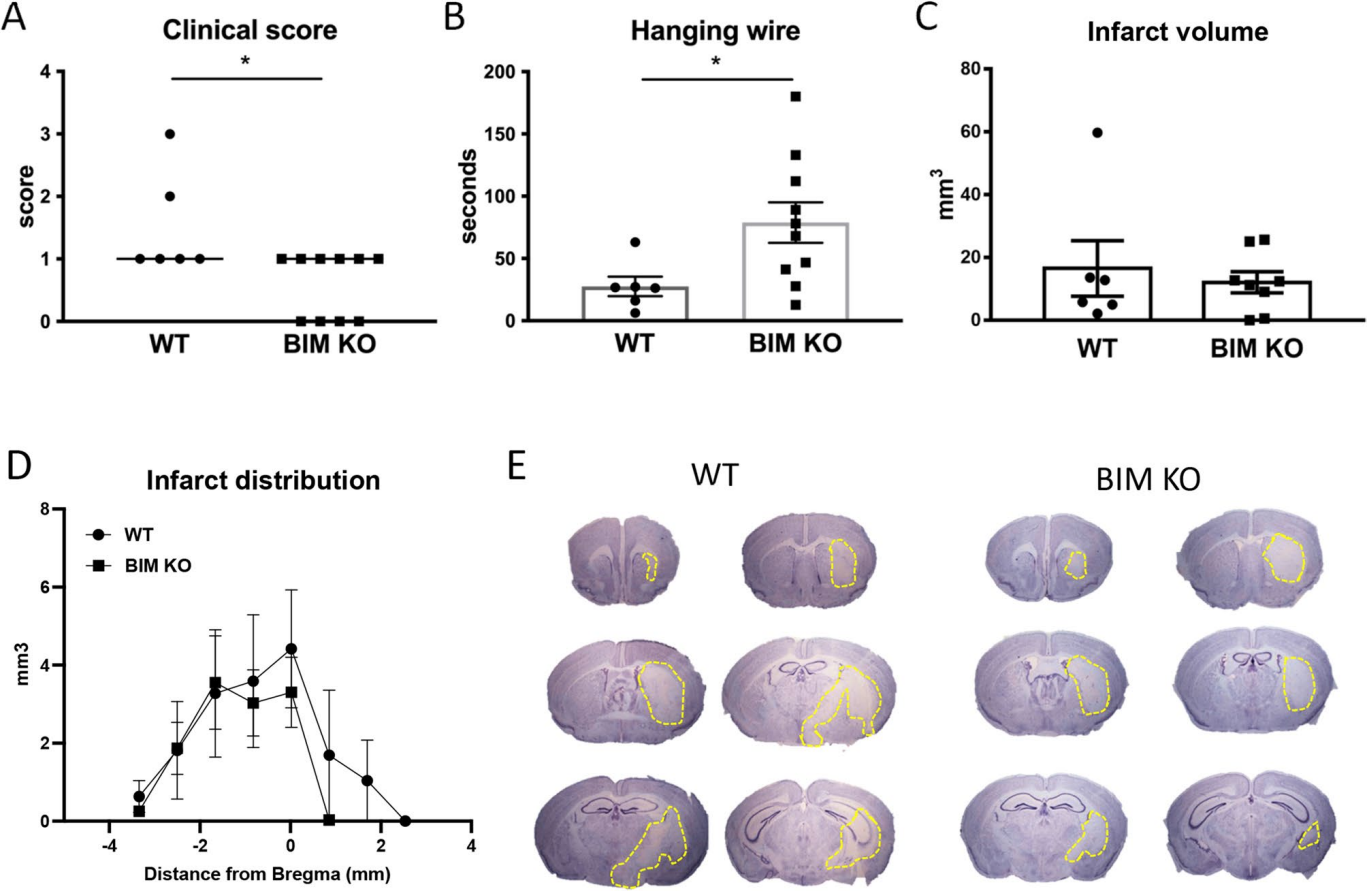
Effect of Bim deletion on functional outcomes and infarct volume. **A** Clinical severity score, **B** hanging wire latency to fall, **C–E** infarct volume and distribution in wild type (WT) and Bim-deficient (BIM KO) mice at 24 h after stroke. **A** Data are presented as median, *P < 0.05 compared with WT, Mann–Whitney test. **B, D** Data are presented as mean ± SEM, *P < 0.05 compared with WT, unpaired *t* test, n = 6–10

Neurological impairment, as measured by median clinical score, was reduced in *Bim*^−/−^ compared to WT mice at 24 h after transient MCAO (Fig. 1A; P < 0.05). Furthermore, latency to fall in the hanging grip test was increased by approximately threefold in *Bim*^−/−^ compared with WT mice (Fig. 1B; P < 0.05). These improvements in functional outcome were evident despite no differences in infarct size or distribution in WT and *Bim*^−/−^ mice (Fig. 1C–E).

### Bim-Deficient Mice Exhibit Less Apoptosis of Immune Cells In Spleen and Thymus After Stroke

**Fig. 2:**
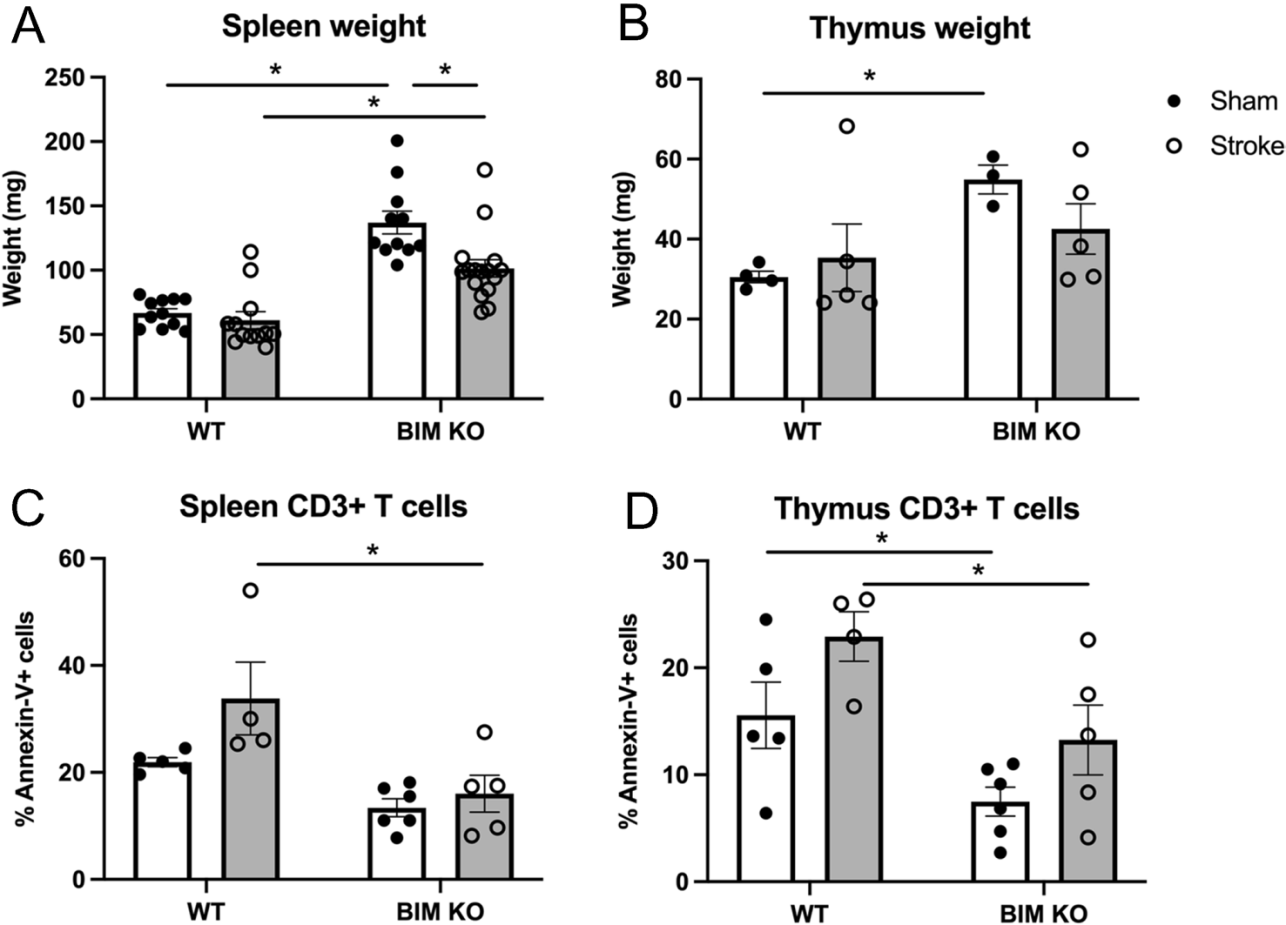
Effect of Bim deletion on apoptosis in peripheral immune organs. **A** Spleen and **B** thymus weight and the number of Annexin V^+^ CD3^+^ T cells in the **C** spleen and **D** thymus in wild type (WT) or Bim-deficient mice 24 h after sham or stroke surgery. Data are presented as mean ± SEM, *P < 0.05 compared with WT, two-way ANOVA with Bonferroni multiple comparisons test, n = 3–16

We found that sham-operated *Bim*^−/−^ mice had larger spleens and thymuses than sham-operated WT mice (Fig. 2A, B), whilst MCAO resulted in a reduced spleen weight in *Bim*^−/−^ mice only (Fig. 2A; P < 0.05). After stroke, there were fewer Annexin V^+^ apoptotic CD3^+^ T cells in spleens and thymuses of *Bim*^−/−^ mice compared with WT mice (Fig. 2C, D).

### Bim-Deficient Mice have Less Infiltration of Immune Cells in the Brain After Stroke

**Fig. 3:**
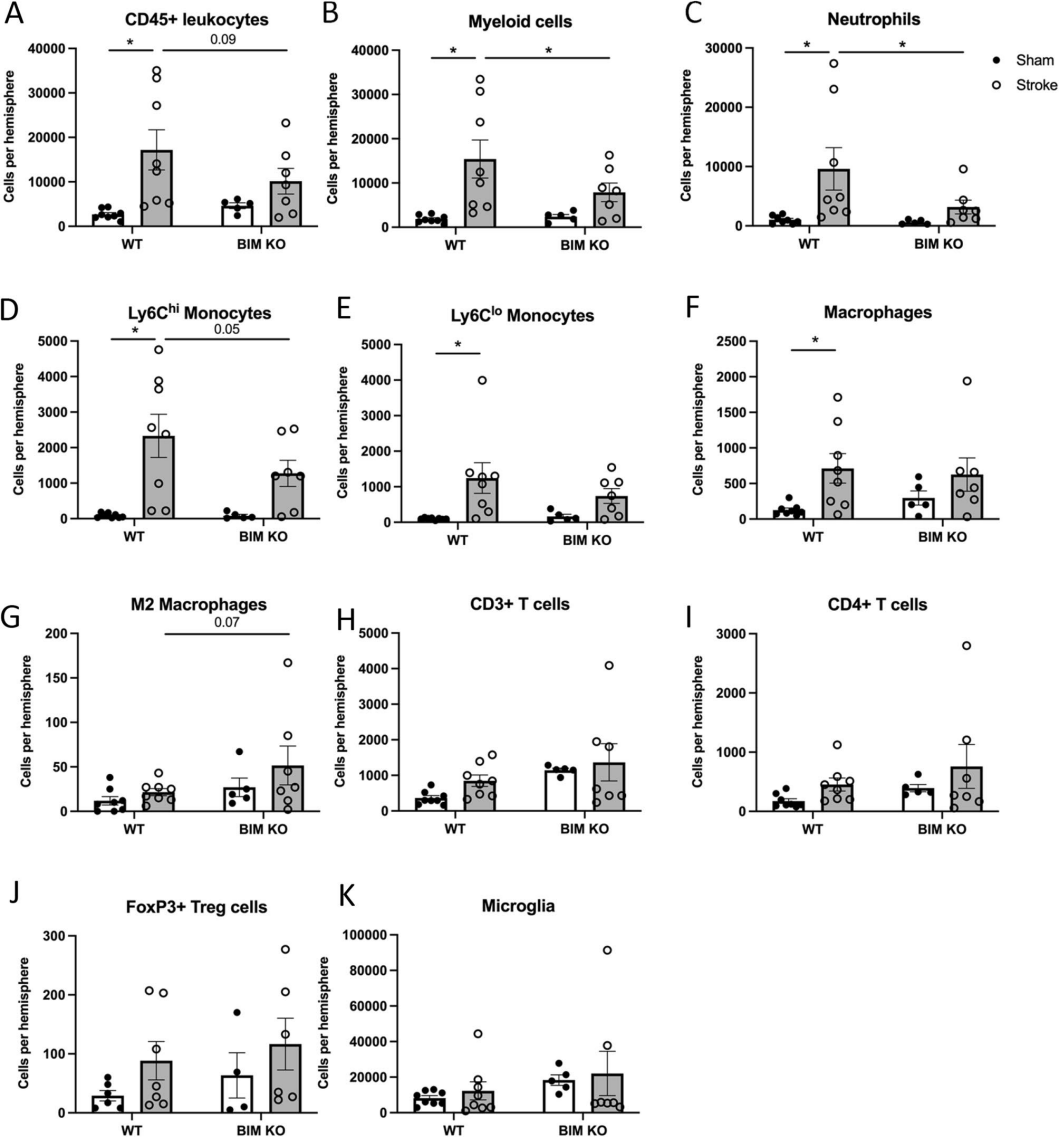
Effect of Bim deletion on leukocytes in the brain after stroke. The number of **A** total leukocytes (CD45^+^), **B** myeloid cells (CD11b^+^), **C** neutrophils (Ly6G^+^), **D** Ly6C^hi^ monocytes, **E** Ly6C^lo^ monocytes, **F** macrophages (F4/80^+^), **G** M2-polarised macrophages (CD206^+^), **H** T cells (CD3^+^), **I** CD4^+^ T cells, **J** T-regulatory cells (FoxP3^+^) and **K** microglia (CD45^med^ CD11b^+^), **C** in the ischemic brain hemisphere at 24 h after stroke. Data are presented as mean ± SEM, *P < 0.05 compared with WT, two-way ANOVA with Bonferroni multiple comparisons test, n = 4–8

In the brain, Bim deficiency did not affect the total number of leukocytes or any individual leukocyte subset in sham-operated mice (Fig. 3). After stroke, the total number of CD45^+^ leukocytes was increased by sixfold in the ischemic hemisphere of WT mice, and this increase tended to be smaller in *Bim*^−/−^ mice (Fig. 3A). Similar profiles, with smaller post-stroke increases in *Bim*^−/−^ mice, were observed for myeloid cells (CD11b^+^) and neutrophils (Ly6G^+^) (Fig. 3B, C). There was also a trend for fewer Ly6C^hi^ monocytes (Fig. 3D) and more M2 (CD206^+^)-polarised macrophages (Fig. 3G) in the brain of *Bim*^−/−^ mice than WT mice after stroke, but no strain-dependent differences in number of Ly6C^lo^ monocytes or macrophages (Fig. 3E, F). Neither surgery nor Bim expression had any effect on the number of CD3^+^ T cells, CD4^+^ T cells or FoxP3^+^ T cells (T-regulatory cells) (Fig. 3H–J). The number of microglia (CD45^med^ CD11b^+^) was similar in all groups (Fig. 3B).

### Bim-Deficient Mice have More Circulating Immune Cells After Stroke

**Fig. 4:**
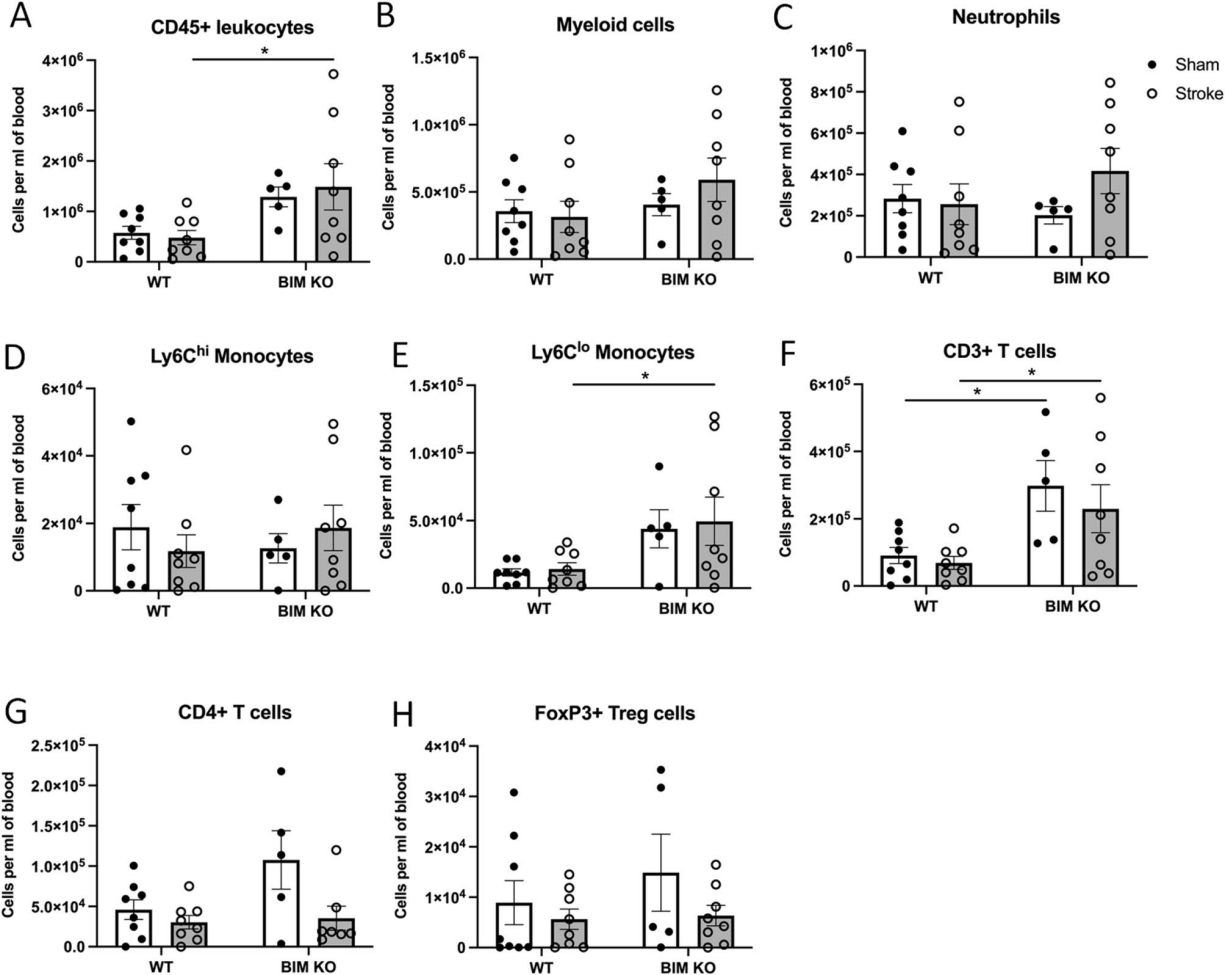
Effect of Bim deletion on circulating leukocytes after stroke. The number of **A** total leukocytes (CD45^+^), **B** myeloid cells (CD11b^+^), **C** neutrophils (Ly6G^+^), **D** Ly6C^hi^ monocytes, **E** Ly6C^lo^ monocytes, **F** T cells (CD3^+^), **G** CD4^+^ T cells and **H** T-regulatory cells (FoxP3^+^) per mL of blood 24 h after stroke. Data are presented as mean ± SEM, *P < 0.05 compared with WT, two-way ANOVA with Bonferroni multiple comparisons test, n = 5–8

In the blood of sham-operated mice, there was a tendency for more CD45^+^ leukocytes in *Bim*^−/−^ than WT mice (Fig. 4A). At 24 h after stroke, there were more circulating CD45^+^ leukocytes in *Bim*^−/−^ mice compared with WT mice (Fig. 4A). There were more circulating Ly6C^lo^ monocytes and CD3^+^ T cells in *Bim*^−/−^ mice compared with WT mice, with no effect of stroke (Fig. 4E, F). Neither stroke nor deletion of Bim affected the number of circulating myeloid cells, neutrophils, Ly6C^hi^ monocytes, CD4^+^ T cells, or FoxP3^+^ T cells (Fig. 4B–D, G–H).

## Discussion

Apoptosis is known to be a key pathological mechanism following stroke. As well as directly affecting the brain parenchyma, apoptosis may influence post-stroke outcomes by regulating immune cell survival. As an important regulator of the pro-apoptotic pathway, it is plausible that Bim may influence stroke outcomes. In this study, we utilised mice deficient in Bim to determine the effect of its expression on outcomes after ischemic stroke. In comparison to WT mice, we found that *Bim*^−/−^ mice had milder functional impairment at 24 h following ischemic stroke despite a similar infarct volume. Bim deficiency also reduced lymphocyte apoptosis in peripheral immune organs and infiltration of select immune cell subtypes into the brain after stroke. Our findings suggest that *Bim*^−/−^ mice have improved functional outcomes after ischemic stroke compared with WT mice, and this may be attributable to reduced immune cell apoptosis and infiltration into the brain.

The ultimate goal of stroke therapy is to improve functional outcomes. Studies have shown that functional improvements after ischemic stroke are not necessarily simply related to a reduced infarct volume (Kim et al., 2014; Zhang et al., 2018). Thus, whilst neuroprotective agents—for example—may be a focus of research efforts to limit infarct growth, a failure to improve functional outcomes will result in limited or no clinical utility. In the present study, we show that genetic deletion of Bim significantly reduces clinical neurological severity score and improves forelimb grip strength compared with WT mice despite no difference in infarct volume or distribution.

Bim plays a key role in regulating leukocyte apoptosis. Bim deletion has been shown to result in increased numbers of neutrophils and T cells in the spleen and thymus (Bouillet et al., 2002; Chougnet et al., 2011; Herold et al., 2014; Villunger et al., 2003). Consistent with these findings, we found that *Bim*^−/−^ mice had enlarged spleens, which was associated with reduced Annexin V^+^ staining of CD3 cells. *Bim*^−/−^ mice also had reduced apoptosis in the thymus after stroke compared with WT mice. The reduced level of T-cell apoptosis in *Bim*^−/−^ mice may contribute to the increased number of circulating T cells that we observed in all *Bim*^−/−^ mice. Therefore, peripheral leukocyte apoptosis may be an important factor in stroke outcomes. Indeed, simvastatin treatment has been shown to reduce post-stroke splenocyte apoptosis and spleen atrophy, which was associated with reduced brain injury and functional impairment (Jin et al., 2013). Thus, preventing splenic atrophy may lead to improved stroke outcomes.

The role of inflammation in the pathogenesis of ischemic stroke is of significant interest. Due to the fact that inflammation occurs over hours to days, and potentially chronically, after the ischemic event, inflammatory mechanisms may represent an opportunity for therapeutic intervention that is much more feasible than the 4.5-h window for clot-buster therapy (Powers et al., 2018). It is important to note that infiltrating immune cells can exert either pro- or anti-inflammatory effects in the brain following the onset of ischemia (Zhang et al., 2021). This complex inflammatory response to ischemic stroke is highlighted by the finding that administration of a broad-specificity chemokine-binding protein is only temporarily effective in delaying inflammation-driven infarct development, indicating that other inflammatory molecules and pathways besides chemokine signalling are involved (Lee et al., 2015). Thus, a clearer understanding of the role of specific immune cell subtypes, chemokines and cytokines is needed to enable the development of immunomodulatory therapies. Consistent with previous studies, MCAO promoted leukocyte infiltration into the brain (Benakis et al., 2016; Chu et al., 2014; Gelderblom et al., 2009; Kim et al., 2014). In transient MCAO, infiltrating immune cells are thought to localise to the infarct core and the peri-infarct region (Beuker et al., 2021). Bim deficiency tended to reduce the overall number of infiltrating cells, and furthermore there were clear changes in the profile of infiltrating cells. In particular, Bim deficiency resulted in fewer infiltrating myeloid cells following stroke, which was primarily due to reduced numbers of neutrophils entering the ischemic hemisphere. Neutrophils are amongst the first cells to enter the brain following an ischemic event and may contribute to worsened outcomes (Chou et al., 2004; Chu et al., 2014; Gelderblom et al., 2009; Huang et al., 2000). Whilst we did not determine whether the infiltrating neutrophils were pro-(N1) or anti-(N2) inflammatory neutrophils, the accompanying reduction in motor deficit would be consistent with fewer infiltrating N1-like neutrophils in *Bim*^−/−^ mice. In addition, the tendency for an increase in infiltrating M2 macrophages is also consistent with a shift to a more anti-inflammatory profile with Bim deficiency. Different profiles of pro- and anti-inflammatory leukocytes in the post-stroke brain have previously been reported to account for different functional outcomes (Chu et al., 2015; Kim et al., 2014).

Whilst we did not observe an increase in overall T cells, T-helper cell or T-regulatory cell numbers in the ischemic hemisphere of WT mice (Brait et al., 2010; Gelderblom et al., 2009; Kim et al., 2014; Yilmaz et al., 2006), there was a trend for these cells to be increased by 2–3fold after stroke. Like neutrophils and monocytes/macrophages, infiltrating T cells have been shown to be critical mediators of ischemic stroke injury (Hurn et al., 2007; Yilmaz et al., 2006). The reason for these conflicting results is currently unclear.

Whilst the absolute number of T cells infiltrating the brain was similar in WT and *Bim*^−/−^ mice, it is possible that these cells have a reduced inflammatory capacity. Indeed, Bim deficiency has been reported to impair T-cell activation and cytokine production (Ludwinski et al., 2009). T cells lacking Bim have a reduced production of several pro- (e.g. IL-6, IFN-γ) and anti- (e.g. IL-4, IL-10) inflammatory cytokines, which have been shown to play critical roles in stroke injury (Zhang et al., 2021). Thus, Bim deficiency may reduce post-stroke impairment via shifting infiltrating immune cells to a less inflammatory phenotype and impairing T-cell function. This modulation of the inflammatory response after stroke may be the underlying cause of improved functional outcomes despite no change in infarct volume.

In conclusion, here we have provided direct evidence that Bim expression is an important factor in neurological outcome following ischemic stroke. Bim deficiency resulted in modulation of the post-stroke immune response leading to less motor and neurological deficit despite no change in infarct size. This beneficial effect of Bim deletion for stroke outcome may be at least partly related to a shift to a more anti-inflammatory response to injury in the ischemic brain. We therefore postulate that Bim may be a potential therapeutic target for ischemic stroke.
